# *LDLR* and *PCSK9* Are Associated with the Presence of Antiphospholipid Antibodies and the Development of Thrombosis in aPLA Carriers

**DOI:** 10.1371/journal.pone.0146990

**Published:** 2016-01-28

**Authors:** Eguzkine Ochoa, Mikel Iriondo, Carmen Manzano, Asier Fullaondo, Irama Villar, Guillermo Ruiz-Irastorza, Ana M. Zubiaga, Andone Estonba

**Affiliations:** 1 Department of Genetics, Physical Anthropology and Animal Physiology, School of Science and Technology, University of the Basque Country (UPV/EHU), Leioa, Spain; 2 Autoimmune Disease Research Unit, Service of Internal Medicine, BioCruces Health Research Institute, Hospital Universitario Cruces, Barakaldo, Spain; Ottawa Hospital Research Institute, CANADA

## Abstract

**Introduction:**

The identification of the genetic risk factors that could discriminate non- thrombotic from thrombotic antiphospholipid antibodies (aPLA) carriers will improve prognosis of these patients. Several human studies have shown the presence of aPLAs associated with atherosclerotic plaque, which is a known risk factor for thrombosis. Hence, in order to determine the implication of atherosclerosis in the risk of developing thrombosis in aPLA positive patients, we performed a genetic association study with 3 candidate genes, *APOH*, *LDLR* and *PCSK9*.

**Material & Methods:**

For genetic association study we analyzed 190 aPLA carriers -100 with non-thrombotic events and 90 with thrombotic events- and 557 healthy controls. Analyses were performed by χ2 test and were corrected by false discovery rate. To evaluate the functional implication of the newly established susceptibility loci, we performed expression analyses in 86 aPLA carrier individuals (43 with thrombotic manifestations and 43 without it) and in 45 healthy controls.

**Results:**

Our results revealed significant associations after correction in SNPs located in *LDLR* gene with aPLA carriers and thrombotic aPLA carriers, when compared with healthy controls. The most significant association in *LDLR* gene was found between SNP rs129083082 and aPLA carriers in recessive model (adjusted *P*-value = 2.55 x 10^−3^; OR = 2.18; 95%CI = 1.49–3.21). Furthermore, our work detected significant allelic association after correction between thrombotic aPLA carriers and healthy controls in SNP rs562556 located in *PCSK9* gene (adjusted *P*-value = 1.03 x 10^−2^; OR = 1.60; 95%CI = 1.24–2.06). Expression level study showed significantly decreased expression level of *LDLR* gene in aPLA carriers (*P*-value <0.0001; 95%CI 0.16–2.10; SE 0.38–1.27) in comparison to the control group.

**Discussion:**

Our work has identified *LDLR* gene as a new susceptibility gene associated with the development of thrombosis in aPLA carriers, describing for the first time the deregulation of *LDLR* expression in individuals with aPLAs. Besides, thrombotic aPLA carriers also showed significant association with *PCSK9* gene, a regulator of LDLR plasma levels. These results highlight the importance of atherosclerotic processes in the development of thrombosis in patients with aPLA.

## Introduction

Antiphospholipid antibodies (aPLAs) are members of a heterogeneous family of immunoglobulins that recognize a variety of phospholipids or proteins that bind to phospholipids. The persistent presence of aPLAs can lead to the development of Antiphospholipid Syndrome (APS), a complex autoimmune disease characterized by venous and/or arterial thrombosis and/or pregnancy morbidity [1,2,3]. As a complex disease, APS is caused by a combination of genetic and environmental factors like some drugs or infections [4,5]. The genetic component involved in the development of APS is still largely unknown but, although there is no published data on familial aggregation, it may be as important as it is for other autoimmune diseases [6,7]. The main cause of death in APS patients is thrombosis, but albeit all APS individuals have aPLAs, only a fraction of APS patients have thrombotic manifestations and moreover, some aPLA carriers are asymptomatic with respect to APS and thrombosis [8,9]. Currently risk factors that discriminate non-thrombotic aPLA carriers from thrombotic aPLA carriers are still largely unknown. Therefore, the identification of the genetic risk factors involved in thrombotic phenotype will improve prognosis of these patients.

Candidate gene association studies and gene expression profiling have identified APS susceptibility genes involved in coagulation, inflammation and innate immune response [10,11,12,13,14,15,16,17,18,19]. However, and despite some experimental evidences connecting atherosclerosis and aPLA, none of these studies have focused their attention on genes related to atherosclerosis in aPLA carriers. It has been proposed that the development of thrombosis is induces by aPLAs through the propagation and amplification of hemostatic, inflammatory and pro-atherogenic responses in absence of physiological regulation [20,21]. Moreover, experimental models of atherosclerosis as well as human studies have described the presence of aPLAs in atherosclerotic plaques [22]. Among the most relevant predictors for arterial thrombosis and atherosclerotic cardiovascular diseases are those that target β2-glycoprotein I (β2GPI), a plasma protein encoded by the *APOH* gene [23,24]. Atherosclerotic plaques show high levels of β2GPI and oxidized low density lipoproteins (oxLDL), both targets of aPLAs, which can bind forming pro-atherogenic complexes [22,25]. These complexes are considered a risk factor to thrombosis and atherosclerosis in patients with an autoimmune background [26]. Low density lipoproteins (LDL) are removed from vessel by low density lipoprotein receptor (LDLR), encoded by *LDLR* gene, and their plasma levels are regulated by proprotein convertase subtilisin/kexin type 9 (PCSK9), a serine protease that promotes degradation of LDLR in liver [27]. Hence, the presence of genetic variants in *APOH*, *LDLR* and *PCSK9* genes could promote pro-atherogenic responses modifying β2GPI and LDL plasma levels [12,28,29,30,31,32].

In this context, our work attempts to determine the implication of atherosclerosis in the risk of developing thrombosis in aPLA positive patients. For this purpose, we designed a candidate gene study with *APOH*, *LDLR* and *PCSK9* genes, performing genetic association studies and gene expression analyses to compare individuals carrying aPLA with and without thrombosis, and healthy controls.

## Materials and Methods

### Samples

All subjects included in this study were Spanish Caucasian individuals. For the case group we collected individuals with persistently positive aPLA at medium-high titers from the Autoimmune Disease Research Unit of Hospital Universitario de Cruces (Barakaldo, Spain) during years 2008–2010. In the control group we included healthy individuals without family history of autoimmune diseases from the Basque Biobank for Research-OEHUN (Spain). The protocols for human subjects’ recruitment and study were approved by the Ethics Committee for Clinical Research of the Basque Country, and by the Ethics Committee for Research and Teaching of the University of the Basque Country (UPV/EHU). Samples and data from patients were provided by the Basque Biobank for Research-OEHUN (www.biobancovasco.org) and were processed following standard procedures with appropriate ethical approval. All subjects were informed about the study design and goals, and signed the informed consent.

The criteria for diagnostic of APS was based on the conclusions of the XI Antiphospholipid Antibodies International Congress held in Sydney in 2004 [2]. This criterion considers that a patient suffers the syndrome when displaying at least one clinical (vascular thrombosis and/or pathology of gestation) and one laboratory criteria: lupus anticoagulant (LA), anticardiolipin antibodies (aCL) and/or anti-β2GPI). The diagnostic criteria for systemic lupus erythematosus (SLE) was developed by the American College of Rheumatology in 1982 and revised in 1997. A patient that shows four or more criteria (facial erythema, discoid lupus, photosensitivity, oral ulcers, arthritis, serositis, renal impairment, altered CNS, blood disorder, immunological disorder, antinuclear antibodies) at some time during the clinical course of the disease is considered to suffer SLE. Antiphospholipid antibodies were considered positive when the patient shows aCL and/or LA. aCL must be present in the serum or plasma in medium or high titers (ie, > 40 GPLU / ml or MPLU / ml, or above the 99th percentile of the reference values of the laboratory; GPLU: IgG phospholipid units; MPLU: IgM phospholipid units) more than twice, with at least 12 weeks apart analysis. Detection was by ELISA standardized [33,34,35]. LA must be present in the plasma on two or more occasions at least 12 weeks apart analyzes. The detection was performed according to the indications of the International Society of Thrombosis and Haemostasis, scientific subcommittee antibody LA / phospholipid-dependent [36,37].

For this genetic association study one hundred and ninety aPLA carriers (aPLA+) and five hundred and sixty-two healthy controls were collected. To be considered in the aPLA+ case group, individuals had to exhibit medium-high aPLA levels on at least two occasions, analyzed twelve weeks apart. The aPLA+ group was subclassified as follows: non-thrombotic (aPLA+/th-, n = 100) and thrombotic (aPLA+/th+, n = 90). In the non-thrombotic group, we included patients exhibiting obstetric complications, patients with systemic lupus erythematosus and medium-high aPLA titers, and asymptomatic individuals with medium-high aPLA levels (S1 Table). The thrombotic group included patients with primary or secondary APS with one or more thrombotic manifestations. Four type of comparisons were carried out: aPLA+ *vs*. healthy controls; aPLA+/th- *vs*. healthy controls; aPLA+/th+ *vs*. healthy controls and aPLA+/th- *vs*. aPLA+/th+. The number and characteristics of individuals included in the study are shown in Table 1. For the gene expression study we analyzed eighty-six aPLA+ individuals, forty-three with thrombotic manifestations (aPLA+/th+), forty-three without thrombotic manifestations (aPLA+/th-), and forty-five healthy controls (Table 1).

**Table 1:** Characteristics of individuals included in the study.

Analysis	Subgroup	N	Gender (%Females)	Age at inclusion (Years)
**Genotyping**	aPLA+	190	73.70%	50.4±14.7
	aPLA+/th–	100	85.00%	50.3±15.1
	aPLA+/th+	90	63.30%	50.5±14.3
	Controls	557	52.90%	43.2±10.4
**Gene Expression**	aPLA+	84	69.10%	51.5±13.9
	aPLA+/th–	41	85.40%	51.0±13.6
	aPLA+/th+	43	53.50%	52.0±14.3
	Controls	45	82.20%	45.8±13.1

### DNA and RNA extraction

Genomic DNA was extracted from whole blood with Flexigen kit (QiagenInc, Valencia, California, USA) at the Basque Biobank for Research-OEHUN. DNA concentration was measured using a NanoDrop Spectrophotometer (NanoDrop Technologies, Inc, Wilmington, DE).

For RNA extraction, fresh peripheral blood mononuclear cells (PBMC) were isolated from heparinized venous blood by centrifugation on a Ficoll-Paque PLUS (GE Healthcare Bio-Sciences, Piscataway, NJ, USA). Total RNA from PBMCs was isolated with TRIzol Reagent (Invitrogen, Carlsbad, CA, USA) followed by RNeasy Mini Kit (Qiagen, Santa Clara, CA, USA). Purified total RNA was retrotranscribed to cDNA using High Capacity cDNA Reverse Transcription Kit (Applied Biosystems, Foster City, CA, USA), according to manufacturer’s instructions.

### Genetic association analysis

A description of the candidate genes, including their physical location and biological functions is shown in Table 2. SNP selection was carried out by SNP Browser software (Applied Biosystems). We included SNPs that could affect function and/or transcription levels: non-synonymous SNPs and SNPs located in regulatory regions such as 5´UTR, 3´UTR, microRNA-binding sites, or transcription factor binding sites. We also included tagSNPs to cover the majority of all common variation in each gene. In total, a panel of 50 SNPs was selected (S2 Table). TaqMan^®^ SNP Genotyping Assays (Applied Biosystems) was used for genotyping. This system uses two allele-specific MGB probes and two PCR primers to provide highly robust and accurate genotyping calls. Data were analyzed by Taqman Genotyper Software (Applied Biosystems).

**Table 2:** Candidate genes included in the study.

Gene Symbol	Gene Name	Gene ID	Cytoband	SNPs	Biological Functions	References
*APOH*	Apolipoprotein H	350	17q23-qter	14	Blood coagulation	[11,21]
					Fibrinolysis	
					Hemostasia	
					Apoptosis	
					Response to wounding	
*LDLR*	LDL receptor	3949	19p13.3	17	Response to steroid hormone stimulus	[26–28]
					Cholesterol homeostasis	
					LDL particle clearance	
*PCSK9*	proprotein convertase subtilisin/kexin type 9	255738	1p32.3	19	Response to hormone stimulus	[23–25]
					Sterol homeostasis	
					Apoptosis	
					Cellular response to stress	
					LDL particle clearance	

All data were quality-filtered according to the default parameters of Haploview software, v.4.3 [38]: SNPs with a call-rate lower than 90%, or those with deviations from Hardy-Weinberg equilibrium (HWE; *p* < 0.01) were excluded. After applying the quality control and excluding monomorphic SNPs, 38 SNPs remained for the association analysis. Allelic and genotypic association analyses were performed with PLINK software, v.1.07 [39], obtaining for each comparison the odds ratio (OR) with the 95% confidence interval (95%CI). Statistically significant results were those with *p*-values < 0.05 after false discovery rate (FDR) correction for multiple testing [40]. To determine statistical power of our analyses we used software CaTS (www.sph.umich.edu/csg/abecasis/CaTS/).

### eQTLs analysis

We searched expression quantitative traits loci (eQTLs) in SNPs with significant *P*-value after correction in any of the subgroups studied. RegulomeDB (http://regulome.stanford.edu/) and Blood eQTL browser (http://genenetwork.nl/bloodeqtlbrowser/) were used to annotate SNPs with known and predicted regulatory elements.

### Gene expression analysis

In the second stage, we evaluated the level of mRNA expression of those genes that showed significant genetic association with the presence of persistently high aPLA titers, and/or with a thrombotic phenotype. Forty-five healthy controls, forty-three thrombotic, and forty-three non-thrombotic aPLA carriers were analyzed by Real-time PCRusing TaqMan^®^ Gene Expression Assay (Applied Biosystems). The reactions were run by triplicate on an ABI 7900HT Fast Real-Time PCR System, using standard cycling conditions. Threshold cycle (Ct) values in relative quantification mode were determined using 7900HT v.2.3 Sequence Detection Systems software (Applied Biosystems). The results were imported onto version 2.0 DataAssist™ software (Applied Biosystems), to determine the normalization factor of each candidate endogenous control genes. The *YWHAZ* gene, whose expression remained unchanged in all samples, was used as endogenous control. To detect statistically significant changes in gene expression between cases and controls, a relative expression software tool (REST) 2009 (Qiagen) was used [41].

## Results

Table 3 summarizes the **s**ignificant allelic associations detected by χ^2^ test after correction by false discovery rate (FDR). Two significant associations were detected; one located in low-density lipoprotein receptor gene (*LDLR*), and the other one in proprotein convertase subtilisin/kexin type 9 gene (*PCSK9*). SNP rs12983082 located in *LDLR* gene showed significant allelic association when comparing aPLA carriers with healthy controls (adjusted *p*-value = 0.0103; OR = 1.60; 95%CI = 1.24–2.06). The comparison of thrombotic aPLA carriers with healthy controls revealed significant allelic association in SNP rs562556 in *PCSK9* gene (adjusted *p*-value = 0.0336; OR = 1.84; 95%CI = 1.28–2.66). Furthermore, genotypic association analyses also showed significant results in *LDLR* gene after correction (Table 4). These associations were evaluated under dominant, additive and recessive models. Thus, the comparison between aPLA carriers and healthy controls revealed significant associations with two SNPs located in *LDLR* gene, rs129083082 and rs1003723, and in both cases the best fitting model was recessive (rs12983082 adjusted *P*-value = 2.55 x 10^−3^; OR = 2.18; 95%CI = 1.49–3.21; rs1003723 adjusted *P*-value = 0.0106; OR = 1.96; 95%CI = 1.34–2.88). The statistical power of this analysis was 99% (MAF 0.59, OR 2.18, recessive model). Besides, SNP rs12983082 in *LDLR* gene also showed significant genotypic association when comparing thrombotic aPLA carriers and healthy controls (adjusted *P*-value = 0.0354; OR = 2.35; 95%CI = 1.42–3.91). Furthermore, with the aim of searching for the genetic factors that could contribute to the onset of thrombotic manifestations within aPLA+ individuals, we also compared thrombotic with non-thrombotic aPLA carriers. This comparison revealed significant allelic and genotypic association of SNP rs562556 located in *PCSK9* before correction for multiple testing (Allelic level: *P*-value = 0.027; OR = 1.972; 95%CI = 1.06–2.80; Genotypic level: *P*-value = 0.032; OR = 1.92; 95%CI = 1.06–3.52). It could be a promising candidate SNP for differentiating thrombotic with non-thrombotic aPLA carriers to be analyzed in a larger sample.

**Table 3:** Significant allelic associations detected.

*SNP# ID*	*Gene*	*Alleles (1/2)*	*Subgroup*	*N*	*MAF*^*a*^	*P-value*_*unadj*_	*P*_*FDR*_^*b*^	*OR (95%CI)*
rs12983082	*LDLR*	C/T	Controls	557	0.472			
			aPLA+	190	0.588	2.71 x 10^−4^	**0.0103**	1.60 (1.24–2.06)
			aPLA+/th+	90	0.597	3.85 x 10^−3^	0.0732	1.66 (1.17–2.35)
			aPLA+/th-	100	0.580	8.45 x 10^−3^	0.3212	1.55 (1.12–2.14)
			aPLA+/th+ vs aPLA+/th-^c^	90	0.597	0.7557	0.8906	1.07 (0.69–1.67)
rs562556	*PCSK9*	A/G	Controls	557	0.189			
			aPLA+	190	0.247	1.89 x 10^−2^	0.3263	1.40 (1.06–1.87)
			aPLA+/th+	90	0.301	8.85 x 10^−4^	**0.0336**	1.84 (1.28–2.66)
			aPLA+/th-	100	0.200	0.7333	0.8607	1.07 (0.73–1.57)
			aPLA+/th+ vs aPLA+/th-^c^	90	0.301	2.72 x 10^−2^	0.8137	1.72 (1.06–2.80)

^a^ MAF, minor allele frequency.

^b^ *P*-values adjusted using FDR [40].

^c^ Comparison of aPLA+/th+ (N = 90) with aPLA+/th- (N = 100).

**Table 4:** Significant genotypic associations detected.

						N(%)				
SNP# ID	Gene	Alleles (1/2)	Subgroup	N	Model	1/1	1/2+2/2	P-value _unadj_^a^	P_FDR_^b^	OR (95%CI)
rs12983082	*LDLR*	C/T	Controls	557		103 (21.3)	380 (78.7)			
			aPLA+	190	Recessive	61 (37.2)	103 (62.8)	6.71 x 10^−5^	**2.55 x 10**^**−3**^	2.18 (1.49–3.21)
			aPLA+/th+	90	Recessive	30 (38.9)	47 (61.0)	9.33 x 10^−4^	**0.0354**	2.35 (1.42–3.91)
			aPLA+/th-	100	Recessive	31 (35.6)	56 (64.4)	4.27 x 10^−3^	0.1271	2.04 (1.25–3.33)
			aPLA+/th+ vs aPLA+/th-^c^	90	Recessive	30 (38.9)	47 (61.0)	0.6599	1	1.15 (0.61–2.17)
rs1003723	*LDLR*	T/C	Controls	557		117 (22.6)	400 (77.4)			
			aPLA+	190	Recessive	58 (36.5)	101 (63.5)	5.56 x 10^−4^	**0.0106**	1.96 (1.34–2.88)
			aPLA+/th+	90	Recessive	27 (36.5)	47 (63.5)	9.33 x 10^−4^	0.1610	1.96 (1.17–3.29)
			aPLA+/th-	100	Recessive	31 (36.5)	54 (63.5)	6.69 x 10^−3^	0.1271	1.96 (1.20–3.19)
			aPLA+/th+ vs aPLA+/th-^c^	90	Recessive	n.d.	n.d.			
rs562556	*PCSK9*	A/G	Controls	557		189 (34.9)	352 (65.1)			
			aPLA+	190	Dominant	77 (43.3)	101 (56.7)	4.65 x 10^−2^	0.2947	1.42 (1.05–2.00)
			aPLA+/th+	90	Dominant	43 (51.8)	40 (48.2)	3.46 x 10^−3^	0.1315	2.00 (1.26–3.19)
			aPLA+/th-	100	Dominant	34 (35.8)	61 (64.2)	0.8722	0.9969	1.04 (0.65–1.64)
			aPLA+/th+ vs aPLA+/th-^c^	90	Dominant	43 (51.8)	40 (48.2)	3.22 x 10^−2^	0.7766	1.92 (1.06–3.52)

^a^ Logistic regression.

^b^ *P*-values adjusted using FDR [40] Significant *P*-values in bold.

^c^ Comparison of aPLA+/th+ (N = 90) with aPLA+/th- (N = 100).

* Allele combinations under recessive model. In Dominant model (rs562556), 1/1 genotype includes homozygotes for allele 2 and 1/2+2/2 column includes the heterozygote plus allele 1 homozygote. n.d, no data.

**Fig 1:**
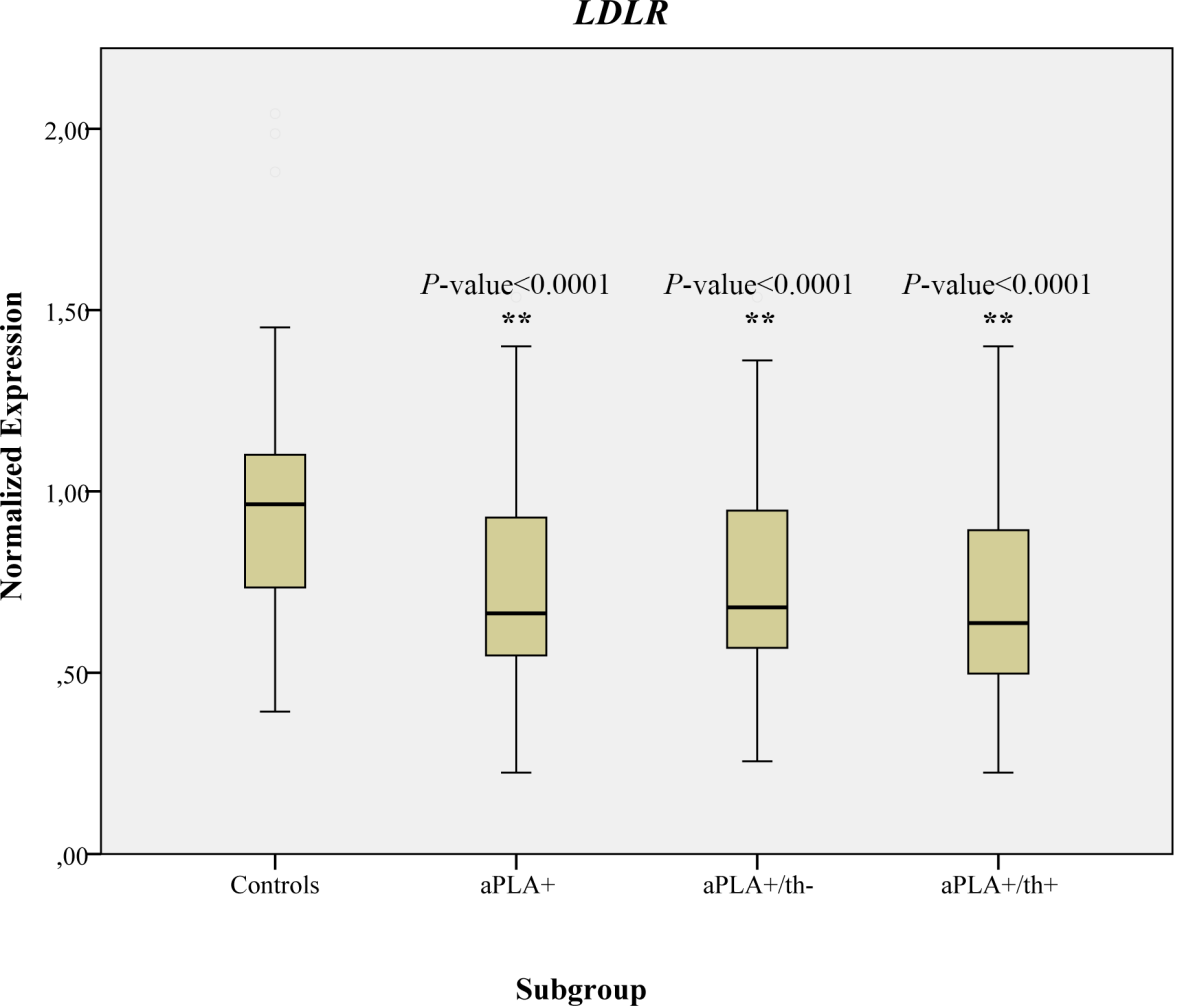
Normalized gene expression of *LDLR* gene in healthy controls, aPLA+, aPLA+/th- and aPLA+/th+ individuals. Two asterisks denote statistically significant differences between case and controls (*P*-value < 0.0001).

Finally, to evaluate if these SNPs could affect gene expression levels, we searched for data in eQTLs databases, RegulomeDB (http://regulome.stanford.edu/) [42] and Blood eQTL Browser (http://genenetwork.nl/bloodeqtlbrowser/) [43]. Thereby, we found significant putative functional effects related with SNP rs12983082 located in *LDLR* gene. RegulomeDB scored this SNP as 3a (being 1 the highest score), which means that it is likely to affect transcription factor binding, any motif and DNase peak. Interestingly, the expression of *LDLR* gene was significantly decreased in aPLA carriers (*P*-value < 0.0001; 95%CI 0.16–2.10; SE 0.38–1.27) in comparison to control group, regardless of thrombotic phenotype, aPLA+/th- (*P*-value < 0.0001; 95%CI 0.14–2.14; SE 0.34–1.27) and aPLA+/th+ (*P*-value < 0.0001; 95%CI 0.17–2.08; SE 0.40–1.27) (Fig 1). *PCSK9* was excluded from further analyses given their undetectable level of expression at the mRNA level in PBMCs derived from all samples.

## Discussion

High aPLA titers are commonly recognized as a thrombosis risk factor, whereby a fraction of antibody carriers will develop the full anti-phospholipid syndrome. Identification of the risk factors that trigger thrombotic manifestations in aPLA carriers should help prevent the development of these symptoms in APS. We searched for susceptibility genes involved in atherogenic processes by a combination of genetic association study and gene expression analyses in aPLA+ individuals with and without thrombosis. Our results have revealed that two genes involved in cholesterol metabolism [44], *LDLR* and *PCSK9*, are associated with the presence of aPLA and with the susceptibility of developing thrombosis among individuals with high aPLA titers.

Low density lipoprotein receptor (LDLR) removes from the vessels low density lipoproteins (LDL), which contain cholesterol esters, phospholipids, triglycerides and apolipoproteins [44], and its deficiencies have been previously related with development of hypercholesterolemia and atherosclerotic lesions [45,46]. The reduced *LDLR* mRNA levels that were observed in the present study in aPLA+ carriers suggest a link between *LDLR* expression and aPLA production in an autoimmunity-prone background. It has been described that loss of murine *ldlr* expression in the context of a pro-autoimmune background leads to immune hyperactivity, characterized by increased activation of B and CD4+ T cells, and to an elevated production of aPLAs against cardiolipin and oxidized LDL [47]. Besides, atherosclerotic plaques in humans commonly exhibit high levels of aPLAs, β2GPI and oxidized LDL (oxLDL), all of them aPLAs targets [22,25]. Whether low *LDLR* expression levels in PBMCs are associated with thrombotic manifestations in patients with aPLAs remains to be clarified.

Our results also detected a significant association between thrombotic aPLA carriers and *PCSK9*, a gene that encodes a regulator of LDLR plasma levels. Besides, this gene is the only one that showed significant associations before correction in the comparison between thrombotic and non-thrombotic aPLA carriers. All these observations suggest that PCSK9 could play a role in the thrombotic phenotype. Unfortunately, *PCSK9* is not expressed at detectable levels in PBMCs and further studies will be necessary to uncover their contribution to thrombosis in patients with aPLAs.

In contrast, we did not find significant genetic association between aPLA carriers and *APOH*, which has been previously studied in this kind of patients due to the hypothesis that some structural defects in β2GPI could be related with the stimulation of autoantibodies production [22]. Yasuda et al. (2005) detected significant associations, but as in the case of our work, those results could not be replicated in independent cohorts [48,49].

It should be stressed that in the present study we included individuals with arterial and venous thrombosis in thrombotic risk group (aPLA+/thr+), and that the arterial thrombosis was the most common (60%). This fact may explain, in part, the strong association found with atherosclerosis-related genes, but this also suggests that OR (odds ratio) levels of significance could be even higher if the study was focused only on arterial thrombosis in patients with aPLA+. In any case, these results support the widely proposed hypothesis of the implication of atherosclerosis in cardiovascular risk for several autoimmune diseases [26,50].

In summary, our results suggest that *LDLR* gene and *PCSK9* gene might play a role in the production of aPLA and in the development of thrombotic APS, although further validation studies will be needed in order to confirm these findings in larger independent cohorts. Moreover, for the translation of these results to the clinical practice it would be useful the study of specific subgroups as the aPLA high risk profile (presence of lupus anticoagulant (LA) and/or persistent aCL at moderate/high titers), the type/number of thrombotic events (arterial/venous) or the exclusive analysis of primary APS in cohort with sufficient sample size.

## Supporting Information

S1 Table Clinical features of aPLA+ individuals. (DOC)

S2 Table SNP selected in the study. (DOC)
