# Supplementary material for: LDLR and PCSK9 Are Associated with the Presence of Antiphospholipid Antibodies and the Development of Thrombosis in aPLA Carriers
Source: PLoS One. 2016 Jan 28;11(1):e0146990. doi: 10.1371/journal.pone.0146990 (PMC4731066; doi:10.1371/journal.pone.0146990)
Supplement: S1 Table — (DOC) [file pone.0146990.s001.doc]

**S1 Table.** Clinical features aPLA+ individuals.

|  | *aPLA+/th+* | | *aPLA+/th-* | | *aPLA* | |
| --- | --- | --- | --- | --- | --- | --- |
| **Diagnostic** | **n** | **%** | **n** | **%** | **n** | **%** |
| PAPS | 63 | 70 | 7 | 7 | 70 | 36.8 |
| SLE-APS | 27 | 30 | 3 | 3 | 30 | 15.8 |
| aPLA+-SLE |  |  | 36 | 36 | 36 | 18.9 |
| aPLA+ asymptom. |  |  | 54 | 54 | 54 | 28.4 |
| Total | 90 |  | 100 |  | 190 |  |

PAPS, primary antiphospholipid syndrome; SLE, systemic lupus erythematosus; APS, antiphospholipid syndrome; aPLA, antiphospholipid antibody; asymptom., asymptomatic.
