# Supplementary material for: LDLR and PCSK9 Are Associated with the Presence of Antiphospholipid Antibodies and the Development of Thrombosis in aPLA Carriers
Source: PLoS One. 2016 Jan 28;11(1):e0146990. doi: 10.1371/journal.pone.0146990 (PMC4731066; doi:10.1371/journal.pone.0146990)
Supplement: S2 Table — (DOC) [file pone.0146990.s002.doc]

**S2 Table.** SNP selected in the study

| **Gene Symbol** | **Gene ID** | **Cytoband** | **Position** | **SNP ID (rs)** | **SNP Change** | **SNP Type** | **MAF** |
| --- | --- | --- | --- | --- | --- | --- | --- |
| *APOH* | 350 | 17q23-qter | 61637158 | rs8178870 | A/T | TagSNP | 0.47 |
|  |  |  | 61638747 | rs1801690 | C/G | missense Trp335Ser | 0.042 |
|  |  |  | 61638858 | rs1558359 | C/T | TagSNP | 0.493 |
|  |  |  | 61641042 | rs1801689 | G/T | missense Cys325Gly | 0.069 |
|  |  |  | 61641054 | rs35449692 | C/T | missense Glu321Lys | 0.014 |
|  |  |  | 61642020 | rs8178858 | C/T | TagSNP | 0.254 |
|  |  |  | 61645860 | rs7212060 | G/T | TagSNP | 0.291 |
|  |  |  | 61647277 | rs8178847 | A/G | missense Arg154His | 0.049 |
|  |  |  | 61648440 | rs8178845 | G/T | TagSNP | 0.471 |
|  |  |  | 61649477 | rs3785617 | A/G | TagSNP | 0.298 |
|  |  |  | 61652626 | rs1801692 | A/G | missense Ser107Asn | 0.022 |
|  |  |  | 61655946 | rs3826358 | C/T | missense Val54Ala | 0.01 |
|  |  |  | 61659480 | rs8073149 | C/T | TagSNP | 0.225 |
|  |  |  | 61673165 | rs8064837 | C/G | TagSNP | 0.474 |
|  |  |  |  |  |  |  |  |
| *LDLR* | 3949 | 19p13.3 | 11041047 | rs8102273 | C/T | TagSNP | 0.343 |
|  |  |  | 11063306 | rs6511720 | G/T | TagSNP | 0.146 |
|  |  |  | 11063516 | rs8104549 | C/G | Regulatory | 0.03 |
|  |  |  | 11066975 | rs8102912 | A/G | TagSNP | 0.229 |
|  |  |  | 11071912 | rs2228671 | C/T | cds-synon Cys27Cys | 0.159 |
|  |  |  | 11074462 | rs13306510 | C/T | missense Pro105Ser | 0.001 |
|  |  |  | 11077561 | rs12983082 | A/C | TagSNP | 0.472 |
|  |  |  | 11083300 | rs11669576 | A/G | missense Ala391Thr | 0.039 |
|  |  |  | 11085181 | rs1003723 | C/T | TagSNP | 0.478 |
|  |  |  | 11085931 | rs2738444 | C/T | Regulatory | 0.103 |
|  |  |  | 11088326 | rs2738446 | C/G | 3´UTR | 0.462 |
|  |  |  | rs688 | 11088602 | C/T | Sinonym | 0.454 |
|  |  |  | rs5925 | 11091881 | C/T | cds-synon Val653Val | 0.458 |
|  |  |  | 11097804 | rs2738456 | C/T | TagSNP | 0.298 |
|  |  |  | 11099473 | rs2738459 | A/C | TagSNP | 0.474 |
|  |  |  | 11102915 | rs6413504 | A/G | TagSNP | 0.482 |
|  |  |  | 11103658 | rs1433099 | A/G | TagSNP | 0.259 |
|  |  |  |  |  |  |  |  |
| *PCSK9* | 255738 | 1p32.3 | 55254336 | rs2479397 | A/T | TagSNP | 0.469 |
|  |  |  | 55258794 | rs2495502 | C/G | TagSNP | 0.492 |
|  |  |  | 55267270 | rs2495499 | A/G | Regulatory | 0.488 |
|  |  |  | 55268332 | rs2479417 | A/G | TagSNP | 0.314 |
|  |  |  | 55276980 | rs28362198 | A/T | 5´UTR | 0.42 |
|  |  |  | 55277238 | rs2479409 | A/G | TagSNP | 0.342 |
|  |  |  | 55278235 | rs11591147 | G/T | missense Arg46Leu | 0.016 |
|  |  |  | 55278256 | rs11583680 | C/T | missense Ala53Val | 0.158 |
|  |  |  | 55281801 | rs10888896 | C/G | TagSNP | 0.249 |
|  |  |  | 55288592 | rs11206514 | A/C | TagSNP | 0.41 |
|  |  |  | 55291340 | rs7552841 | C/T | TagSNP | 0.468 |
|  |  |  | 55296390 | rs28362261 | A/G | missense Asn425Ser | 0.04 |
|  |  |  | 55296443 | rs28362263 | A/G | missense Ala443Thr | 0.04 |
|  |  |  | 55296825 | rs562556 | A/G | missense Val474Ile | 0.189 |
|  |  |  | 55297430 | rs568052 | C/T | TagSNP | 0.33 |
|  |  |  | 55297901 | rs28362270 | A/G | missense His553Arg | 0.01 |
|  |  |  | 55299810 | rs28362277 | A/C | missense Gln619Pro | 0.01 |
|  |  |  | 55301775 | rs505151 | A/G | missense Gly670Glu | 0.11 |
|  |  |  | 55301803 | rs28362286 | A/C | nonsense Cys679OPA | 0.01 |
